# Pigmented Squamous Cell Carcinoma In Situ: Report of a New Case and Review of the Literature

**DOI:** 10.4317/jced.54053

**Published:** 2017-11-01

**Authors:** Fabiana Martins, Florence-Zumbaio Mistro, Sergio Kignel, Michelle Palmieri, Alan-Motta do Canto, Paulo-Henrique Braz-Silva

**Affiliations:** 1 PhD, MDS, DDS, School of Dentistry, University of Santo Amaro, Sao Paulo, SP, Brazil; 2 MDS, DDS, Division of Oral Diagnosis, Department of Dentistry, Herminio Ometto Fondation – UNIARARAS, Araras, SP, Brazil; 3 PhD, MDS, DDS, Division of Oral Diagnosis, Department of Dentistry, Herminio Ometto Fondation – UNIARARAS, Araras, SP, Brazil; 4 MDS, DDS, Division of General Pathology, Department of Stomatology, School of Dentistry, University of São Paulo, Sao Paulo, SP, Brazil; 5 PhD, MDS, DDS, Laboratory of Virology, Institute of Tropical Medicine of Sao Paulo, University of Sao Paulo, Sao Paulo, SP, Brazil

## Abstract

Pigmented squamous cell carcinoma in situ (PSCCIS) is very rare, being clinically described as a pigmented lesion with histological characteristics of an in-situ carcinoma presenting pigmentation within neoplastic cells. A 50-year-old Afro-descendant man came for clinical evaluation of a painful black and red lesion located on the right aspect of the oropharyngeal isthmus. After incisional biopsy, the resulting sample was described as a pigmented squamous cell carcinoma in situ, a diagnosis further confirmed by immunohistochemical analysis. Treatment consisted in total excision of the lesion, and no recurrence was observed after a 30-month follow-up. Clinicians and pathologists should be aware of PSCCIS as a differential diagnosis of melanoma, a lesion which significantly increases the morbidity and mortality rates among these patients.

** Key words:**Pigmented squamous cell carcinoma in situ; oropharyngeal mucosa; immunohistochemistry.

## Introduction

Pigmented squamous cell carcinoma in situ (PSCCIS) in oral and oropharyngeal mucosa is a very rare lesion, being first described in 1974 as an oral squamous cell carcinoma (OSCC) of the tongue and clinically characterized as an asymptomatic pigmented lesion (1). Histologically, PSCCIS presents characteristics of a usual carcinoma in situ - a squamous epithelium with severe epithelial dysplasia and drop-shaped rete ridges showing mitoses and keratin pearls, with dendritic melanocytes within the epithelium (1-3). The differential diagnosis includes nevi, melanoma, lentigo, purpura, ephelis, dermatofibroma and Mongolian spot (1-3). Since the first description of the lesion in the literature, only 3 cases have been reported in English (3).

## Case Report

**Figure 1:**
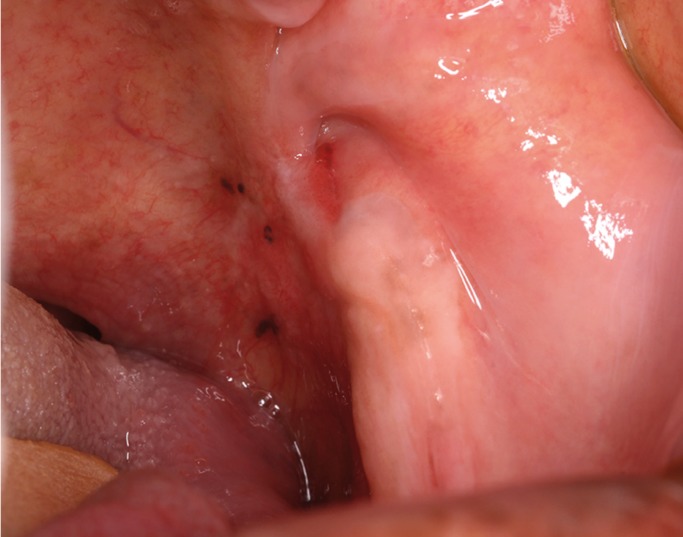
Clinical aspects: presence of dark spots and erythema located in the isthmus.

An African-descendant man in his 50s was referred to our clinic for evaluation of a painful, black and red lesion located on the oropharyngeal isthmus. Medical records had revealed history of stroke 8 months earlier and hypertension. With regard to habits and addictions, the patient reported being a chronic smoker for 30 years, but he stopped after a vascular event. Oral pain and bleeding in the past 6 months were the major complaints reported during interview. Intraoral examination showed three circular black spots surrounded by erythema, all located on the soft palate on the left side and extending to the oropharyngeal mucosa (Fig. 1). Each lesion measured approximately 3 mm in diameter. Incisional biopsy of the area was performed and the resulting sample was fixed with 10% neutral buffered formalin before being cut into 4-μm sections and stained with hematoxylin-eosin (H&E). Immunohistochemistry assay was performed in order to rule out melanoma. The immunohistochemical profile and antibodies used are listed in  Table 1.

**Table 1:**
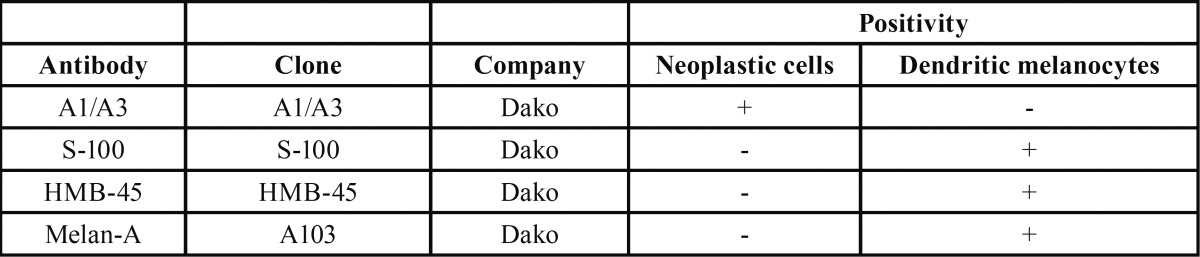
Antibodies used in the immunohistochemical analysis.

**Figure 2:**
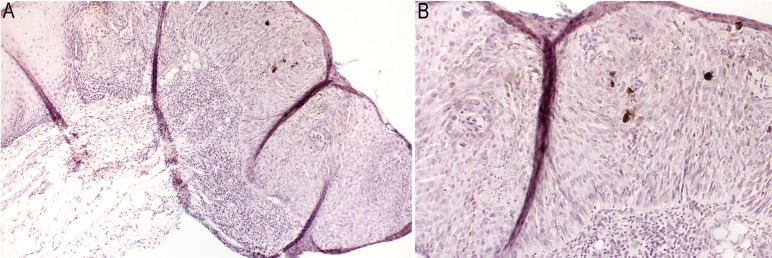
A,B) Histopathological image; conventional characteristics of in situ carcinoma, with dendritic pigmented cells observed in the middle portion of the epithelium [H&E; original magnification of 100x (A) and 200x (B)].

Histological analysis revealed dysplastic cells, showing basaloid appearance all over the epithelium. All the epithelial layers presented irregular stratification and drop-shaped rete ridges, with an increase of mitotic figures and atypical keratinocytes, all characteristic of squamous cell carcinoma in situ (Fig. 2A). Presence of abundant melanin pigment surrounding dysplastic dendritic-shaped cells was mostly seen in upper layers of the epithelium (Fig. 2B).

**Figure 3:**
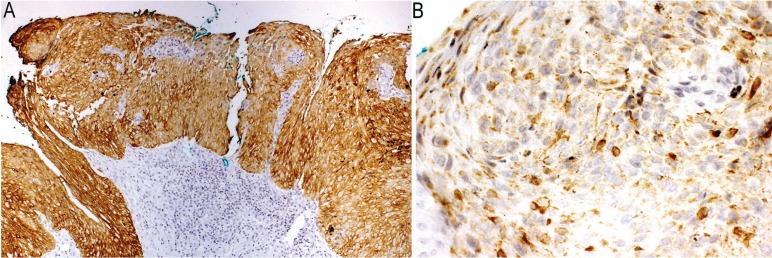
A,B) Strong positivity for pan-cytokeratin A1- A3 (original magnification of 100x) (A); Melan-A expression in normal melanocytes (original magnification of 400x) (B).

Immunohistochemical analysis (Fig. 3) showed that dysplastic and neoplastic cells were positive for pan cytokeratin A1-A3 (Fig. 3A), demonstrating the epithelial origin of the malignancy. The diagnosis of negative staining for S-1OO, HMB-45 and Melan-A proteins within neoplastic cells ruled out the diagnostic possibility of melanoma (Fig. 3B).

After the final diagnosis, the patient was referred to a cancer hospital for treatment, which consisted of total surgical removal of the lesion, including its margins. Recurrence was not observed after a 30-month follow-up.

## Discussion

Pigmented squamous cell carcinoma is a rare variant of squamous cell carcinoma, which is clinically differentiated from melanin pigmentation, pigmented nevus (associated or not with syndromes) and malignant melanoma and is characterized histologically as a typical SCC in combination with non-neoplastic melanocytes within the lesion (3-5).

Both pigmented squamous cell carcinoma and PSCCIS have been described in the conjunctiva and esophagus as well as in the, genital and oral mucosa, but such lesions are rare as they represent less than 5% of all neoplasms in these anatomical sites (5-7).

Only 16 cases of pigmented OSCC were reported in oral mucosa (4), among these two were described as PSCCIS (1,2). To our knowledge, this is the fourth case of PSCCIS in oral/oropharyngeal mucosa described in the literature, and differently from earlier reports (1-3), our patient did not present white patches associated with the pigmented lesion.

According to our review of the literature, our case is the first one identified in a posterior site in the oral cavity. The oropharyngeal isthmus can present clinical and histological characteristics common to those found in the esophagus (5). Clinically, the present case is characterized by well-defined small black dots in the oropharyngeal mucosa, whereas in the only case involving the esophagus as reported in the literature, the authors described their finding as a “small black spot” (5). We did not observe any association with a white patch in this case.

Although this lesion was first described as asymptomatic (1,8), our patient’s major complaint was pain and bleeding, which can be attributed to the fact that the patient clinically presented atrophic areas within the black spots. Pain was reported in the third case of PSCCIS in oral mucosa as available in the literature, haphalgesia during intraoral examination (3).

Other authors attribute the occurrence of PSCCIS to ethnic differences, which can be seen in African-origin individuals with different patterns of melanosome distribution within the cytoplasm (2). The PSCCIS cases described in the literature were observed in afro-descendent individuals in three reports (including the present case), and in one report, this lesion was observed in an Asian patient. This corroborates the fact that in these individuals, there is an increased physiological accumulation of melanin in the gingival tissues (8).

In normal tissues, melanocytes act to protect keratinocytes from injuries (e.g. UV exposure) and to produce melanin pigments, representing about 3-5% of the cell population (9). Melanin accumulates in small granules called melanosomes, which can be present in varying numbers within melanocytes, Langerhans cells, macrophages and neoplastic squamous cells (11).

Several authors describe the origin and pathogenesis of melanocytosis in cases of cancer, such as adenocarcinomas, calcifying epithelioma, exocrine gland tumors, and seborrheic keratosis, among others (9). Some authors discuss the capacity of malignant cells to produce cytokines, thus inducing melanocyte proliferation and consequently melanin production (7). Satomura *et al.* demonstrated a higher expression of endothelin-1 and stem cell factor in the neoplastic squamous cells of a case of pigmented squamous cell carcinoma compared to non-pigmented lesions (12).

The immunohistochemistry study was performed to evaluate the behavior of the lesion and to rule out any diagnostic possibility of melanoma. The antibodies S-100 and HMB-45 are commonly used for diagnosis of melanoma, with the former being used to detect melanocytes lacking melanin and the latter being mostly observed in melanocytes presenting melanogenic activity (3).

In our case, the positivity for HMB-45 was additionally found in proliferating melanocytes and upper layers of the epithelium with and without atypia, as demonstrated in esophagus (5).

## Conclusions

Despite the rarity of this malignancy, the histopathological diagnosis of oral pigmented carcinoma in situ should be taken into consideration to rule out melanoma diagnosis, a lesion with poor prognosis in comparison to a carcinoma in situ.
